# Advancing 3D Spheroid Research through 3D Scaffolds Made by Two-Photon Polymerization

**DOI:** 10.3390/bioengineering11090902

**Published:** 2024-09-09

**Authors:** Eglė Vitkūnaitė, Eglė Žymantaitė, Agata Mlynska, Dovilė Andrijec, Karolina Limanovskaja, Grzegorz Kaszynski, Daumantas Matulis, Vidmantas Šakalys, Linas Jonušauskas

**Affiliations:** 1 Vital3D Technologies, Saulėtekio al. 15, LT-10224 Vilnius, Lithuania; ev@vital3d.eu (E.V.); da@vital3d.eu (D.A.); kl@vital3d.eu (K.L.); vs@vital3d.eu (V.Š.); 2 Laboratory of Immunology, National Cancer Institute, P. Baublio g. 3B, LT-08406 Vilnius, Lithuania; eglezyman@gmail.com (E.Ž.); agata.mlynska@gmail.com (A.M.); 3 Life Sciences Center, Vilnius University, Sauletekio 7, LT-10257 Vilnius, Lithuania; 4 Department of Chemistry and Bioengineering, Vilnius Gediminas Technical University, Saulėtekio al. 11, LT-10223 Vilnius, Lithuania; 5 Department of Biothermodynamics and Drug Design, Institute of Biotechnology, Life Sciences Center, Vilnius University, Sauletekio 7, LT-10257 Vilnius, Lithuania; daumantas.matulis@bti.vu.lt

**Keywords:** spheroids, scaffold, 3D printing, biofabrication, cell cultures, direct laser writing

## Abstract

Three-dimensional cancer cell cultures have been a valuable research model for developing new drug targets in the preclinical stage. However, there are still limitations to these in vitro models. Scaffold-based systems offer a promising approach to overcoming these challenges in cancer research. In this study, we show that two-photon polymerization (TPP)-assisted printing of scaffolds enhances 3D tumor cell culture formation without additional modifications. TPP is a perfect fit for this task, as it is an advanced 3D-printing technique combining a μm-level resolution with complete freedom in the design of the final structure. Additionally, it can use a wide array of materials, including biocompatible ones. We exploit these capabilities to fabricate scaffolds from two different biocompatible materials—PEGDA and OrmoClear. Cubic spheroid scaffolds with a more complex architecture were produced and tested. The biological evaluation showed that the human ovarian cancer cell lines SKOV3 and A2780 formed 3D cultures on printed scaffolds without a preference for the material. The gene expression evaluation showed that the A2780 cell line exhibited substantial changes in *CDH1*, *CDH2*, *TWIST*, *COL1A1*, and *SMAD3* gene expression, while the SKOV3 cell line had slight changes in said gene expression. Our findings show how the scaffold architecture design impacts tumor cell culture 3D spheroid formation, especially for the A2780 cancer cell line.

## 1. Introduction

For many years, two-dimensional (2D) cell culture models in vitro and animal models in vivo have been regarded as the gold standard in cancer research. While 2D cell cultures offer simplicity, cost-effectiveness, and well-established methods for studying cancer cell behavior, they fall short in accurately replicating the intricate structure of native tumor tissue. These cultures cannot mimic the biological, chemical, and mechanical cues present in the three-dimensional architecture of a primary tumor [1,2]. Cancer cells in a 2D cell culture environment adjust their morphology and induce rearrangements in their cytoskeleton, acquiring artificial polarity. One of the main factors impacting their poor success is the inadequacy of the preclinical 2D culture models for accurately replicating the tumor microenvironment (TME). The TME is a complex system incorporating both cellular elements, such as endothelial cells, fibroblasts, mesenchymal stem cells, and endothelial cells, and non-cellular components, including the extracellular matrix (ECM), growth factors, and cytokines, all of which are impactful in cancer development and progression [3,4]. Two-dimensional cell cultures’ applicability to cancer research is limited due to a lack of specific cell–cell and cell–extracellular matrix (ECM) interactions, as well as the unlimited access to nutrients, oxygen, and metabolites, leading to altered gene and protein expression [5]. On the other hand, animal models provide a similar in vivo environment and accurately represent disease for research in which cancer cells or small tissue fragments from the primary tumor are transplanted. Still, the use of animal models has its limitations, such as significant costs and notable limitations in terms of controllability, reproducibility, and design flexibility [6,7].

Recently, 3D cell culture models have gained considerable attention in cancer research. They replicate several characteristics of primary tumor tissue and bridge the gap between 2D cell cultures and intricate animal models [8]. Therefore, the ongoing development of 3D cell culture systems using engineered scaffolds holds promise in replicating the complexity of in vivo tumors, as these cell models offer cell heterogeneity, hypoxia, growth kinetics, and gene expression patterns that closely represent primary tumor tissue [9]. These characteristics position 3D-scaffold-based cancer cell models as promising platforms for testing drug delivery, exploring pluripotency and self-renewal, studying tumor microenvironment interactions, and identifying predictive biomarkers for potential use in future clinical cancer treatments [10]. Three-dimensional cell culture systems generally can be divided into two different approaches—scaffold-free and scaffold-based. The scaffold-free method refers to culturing and forming cells in a 3D structure without an external matrix or scaffold. This allows for more accurate direct cell–cell interactions, physiological responses, and spatial organization, which results in denser spheroids [11]. On the other hand, the scaffold-free method has its limitations in providing mechanical support, shape control, and reproducibility compared to the scaffold-based method. Moreover, the current challenges in creating heterotypic cell spheroids emphasize the unmet need for advanced technologies to model the TME [12]. The scaffold-based cell 3D culturing strategy relies on cells embedding into a polymer-based scaffold to form a 3D architecture. The scaffolds are the key components that provide a 3D environment for cells to grow and interact with each other and their surroundings [13].

Scaffold-based 3D culture fabrication techniques range from 3D-extrusion-based printing to electrospinning [14]. The traditional methods have considerable difficulties in accurately reproducing complex 3D computer-aided design (CAD) models and precisely defining the scaffold geometry in the sub-cellular μm range. Only 3D structures made with such precision can truly mimic the finely nuanced ECM. Additionally, printing technology has to be fast enough to produce statistically relevant numbers (from tens to hundreds) of structures for bio-experiments. In the process of scaffold microfabrication, key design factors include the matrix architecture, pore size and shape, mechanical stability versus void volume, surface properties, and degradation rate [15]. The scaffold must offer enough mechanical strength and stiffness to maintain structural integrity throughout development and cell culture. The scaffold’s architecture should also promote initial cell attachment and facilitate the mass transfer of metabolites while providing ample space for cell proliferation [16]. Among the advanced techniques for fabricating these intricate structures, two-photon polymerization (TPP) stands out due to its precision and versatility [17]. TPP is a laser-based additive manufacturing process based on optical nonlinear absorption for inducing the polymerization or crosslinking of photopolymerizable materials. Due to the nonlinear nature of the process, light–matter interaction is confined only to the focal spot of the focusing optic, making said interaction highly confined and giving it minimal collateral effects. This allows the creation of highly detailed and complex 3D micro- and nanostructures with a sub-micrometer resolution. The advantages of TPP are well understood, resulting in the technology having been applied in a plethora of fields [18,19,20,21,22]. Recently, the main interest in the field has shifted towards exploiting TPP’s capabilities in biofabrication [23,24,25,26]. Here, it is possible to leverage both its resolution and 3D capabilities, as well as exploiting vast options with regard to the materials, which include, but are not limited to, biopolymers, hydrogels, acrylates, elastomers, epoxies, and hybrid materials [27,28]. Unsurprisingly, it has already been demonstrated that TPP is excellent for 3D biomedical structure fabrication and plays a crucial role in the engineering of the cell culture matrices needed to mimic the natural ECM-based microenvironment properly [29].

**Figure 1:**
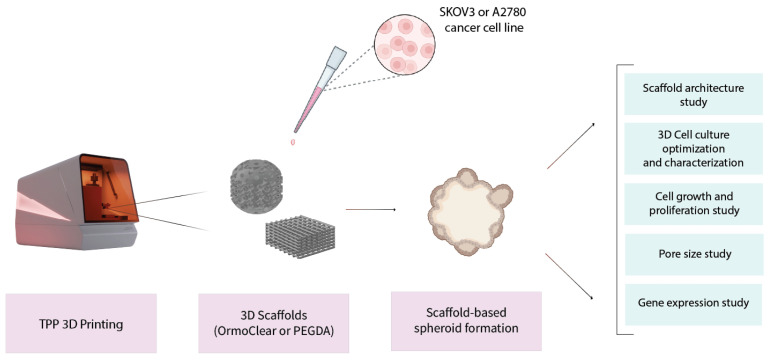
Schematic illustrating study design. First, 3D scaffolds were produced using TPP technology. Then, the structures were seeded with two different human ovarian cancer cell lines—SKOV3 and A2780. Spheroid formation on the scaffolds conformed to and allowed the determination of various peculiarities related to cell line growth on 3D scaffolds, related to the geometry’s impact, cell–material interactions, and gene expression.

This study aims to address the current knowledge gap in cell culture development by exploring cancer cell spheroid formation on 3D scaffolds printed via TPP. As mentioned, 2D cell cultures, although crucial in scientific research, are far from being the optimal tool for carrying out cellular studies. The traditional models lack the proper architectural, mechanical, and biochemical cues that make up the natural tumor microenvironment [30]. This, in turn, influences the gene expression and cellular behavior to provide data that contain inaccuracies when compared to naturally occurring tumors. Thus, our goal was to determine to what extent TPP-made 3D scaffolds can support cancer spheroid formation and unveil possible fabrication-related nuances. A schematic illustrating the study design is shown in Figure 1. We produced two types of scaffolds with distinctly different architectures, geared towards either fast and simple manufacturing or bio-mimicking complex internal geometry. The materials chosen for this study were the hybrid organic–inorganic photopolymer OrmoClear and the hydrogel PEGDA, with the first representing excellent structurability and mechanical properties and the second option being more biocompatible. Subsequently, cancer cell lines were cultured on the scaffolds produced and allowed to proliferate, aiming to determine how the printing materials promote cancer cell growth in vitro. The scaffolds were characterized with SEM, followed by biological evaluation. The scaffolds were seeded with two different human ovarian cancer cell lines—SKOV3 and A2780. Real-time qPCR was used to profile the gene expression. We showed that the application of TPP-produced 3D scaffolds facilitates the growth of cancer cell spheroids. This study shows that a more complex geometry of the scaffold is favorable for 3D cell culture formation and offers a new understanding of how cells interact with the scaffold architecture.

## 2. Materials and Methods

### 2.1. Fabrication of 3D Scaffolds

In this study, two polymers, OrmoClear (Microresist Technology, GmbH) and PEDGA (Mn 700, Sigma Aldrich, Burlington, MA, USA) with 1% of the photoinitiator 2-Hydroxy-4’-(2-hydroxyethyl)-2-methylpropiophenone (Igracure 2959, Sigma Aldrich), were used to print the 3D scaffolds. The hybrid organic–inorganic OrmoClear was chosen due to its wide use and significant chemical and thermal stability. One of the key criteria for choosing the OrmoClear and PEGDA materials was that these families of photopolymers have been explored in depth in previous works. This includes extensive general biocompatibility studies that show their excellent biocompatibility. For this reason, we decided not to perform any additional biocompatibility evaluation in this work. Namely, OrmoClear-style hybrid organic–inorganic photopolymers have been shown to be suitable for cell growth without altering their growth or adhesion properties [31,32]. Using PEGDA with the IGR2959 photoinitiator is especially popular in biological applications due to its super biocompatibility with cells [33,34,35]. More information on the general mechanical properties of these materials can be found in the literature [36,37,38]. The printed samples were developed with 4-methyl-2-pentanone (Sigma Aldrich) for 2 min and rinsed with isopropanol (Sigma Aldrich).

**Figure 2:**
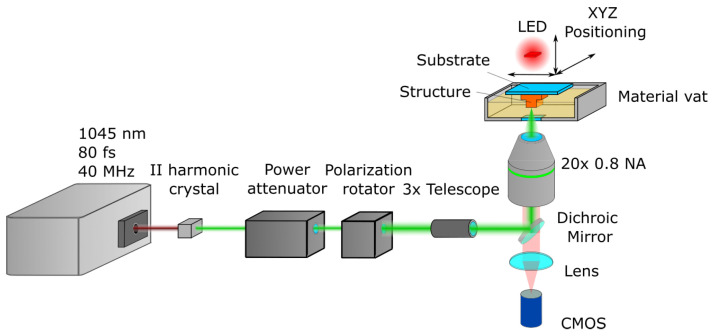
Schematic of laser printing setup used in this work.

A custom laser setup based on a simplified Vital Light 3D workstation was employed for printing (Figure 2). The light source was the femtosecond laser “Biolit 2” (Litilit, Vilnius, Lithuania), emitting 80 fs pulses at a 40 MHz repetition rate. Then, the light was directed at a II harmonic crystal, turning fundamental 1045 nm radiation into 522 nm, followed by a power attenuator “LPA” (Optogama, Vilnius, Lithuania), a polarization control unit “MRO” (Optogama), and a 3× beam expander (Optogama). The beam was expanded to fill the full aperture of the objective lens. A dichroic mirror then directed the laser beam to the objective lens (20× 0.8 NA), which focused it on the sample. The sample was then submerged into a material vat and positioned in 3D using mechanical stages, i.e., the sample moved in relation to the focal point. The structures were fabricated on a 26 mm × 26 mm × 1 mm glass substrate. The setup also had an integrated LED, lens, and CMOS camera (Levenhuk, Prague, Czech Republic), which allowed monitoring of the printing process in situ in real time. The samples were visualized with MRCL700 3D Imager Pro (Microqubic AG, Zug, Switzerland). The morphology of the 3D-printed scaffolds was characterized using a scanning electron microscope (TM-3000, Hitachi, Chiyoda, Tokyo) The samples were not additionally prepared for SEM.

### 2.2. Cell Lines

The human ovarian cancer cell line SKOV3 was obtained from the American Type Culture Collection (ATCC). The human ovarian cancer cell line A2780 was obtained from the European Collection of Cell Cultures (ECACC cat no. 93112517). The A2780 cell line was cultured in 1640 medium (Gibco™, cat. no. 11875093), supplemented with 10% fetal bovine serum (FBS) (Gibco™, cat. no. 16140071) and 1% antibiotics (PS) (Gibco™, cat. no. 15140122), while SKOV3 was cultured in DMEM medium (Gibco™, cat. no. 11965092) supplemented with 10% FBS and 1% PS. The cells were maintained at 37 °C in a humidified atmosphere at 5% CO_2_ and were regularly passaged after they reached confluence. Cell viability was evaluated by performing a Trypan black (Gibco™, cat. no. 15250061) exclusion test.

### 2.3. Cell Culture

The scaffolds were prepared by disinfecting them with 70% ethanol and leaving them under UV light for 20–30 min. After disinfection, the scaffolds were detached and placed in the wells of a 96-well plate, which was prepared following protocol [39], and submerged in the cell-culturing media supplemented with 10% FBS and 1% PS for 1 h. The cells were seeded at density of 7000–14,000 cells per well in 200 μL ofsupplemented culture medium. The cell cultures were maintained at 37 °C in a humidified atmosphere at 5% CO_2_ for 7 days. The 3D cell cultures without the scaffold were made following protocol [39], and 14,000 cells were seeded per well. Cell cultures were observed and imaged every day using an OPTIKA ITALI IM-5 series microscope, while image analysis was performed by employing Fiji, an image processing package.

### 2.4. Evaluation of Gene Expression by Real-Time qPCR

Total RNA was extracted from the cell culture samples on day 7 by TRIzol Reagent (Invitrogen™, Waltham, MA, USA, cat. no. 15596026) following the manufacturer’s protocol. cDNA was obtained by subjecting 500 ng of RNA from each sample to reverse transcription using the Maxima First Strand cDNA Synthesis Kit (Thermo Fisher Scientific, Waltham, MA, USA cat. no. K1642), as described in the accompanying protocol. qPCR was performed in triplicate on an Azure CieloTM 3 Real-Time PCR System. The reaction volume of 10 μL contained 5 μL of Maxima SYBR Green qPCR Master Mix 2X (Thermo Fisher Scientific, cat. no. K0241), 2.5 μL of the 0.8 μmol/L sequence-specific mix of the forward and reverse primers from Table 1, 1 μL of the cDNA reaction product, and 1.5 μL of nuclease-free water. The reaction was started for 5 min at 95 °C and continued with 40 cycles of 10 s of denaturing at 95 °C and 30 s of annealing/extension at 60 °C. The expression levels of the selected genes were evaluated, using the Ribosomal Protein L13a coding gene (RPL13a) as the normalizing gene. The experiment was repeated twice. The analysis was performed with the Azure CieloTM 3 Real-Time PCR System software (version 1.0.0.300.).

## 3. Results

### 3.1. 3D Printing of Scaffolds

**Figure 3:**
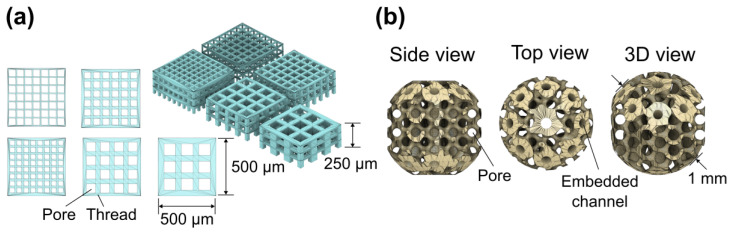
Three-dimensional models of basic (**a**) and spherical (**b**) scaffolds showing the main components and dimensions.

Two scaffold 3D designs were prepared for printing and biological evaluation studies. The first design, which we refer to as the basic scaffold, had cubic unit cells and was 500 × 500 × 250 μm in overall size (Figure 3a). The cubic scaffold was chosen due to the simplicity of its processing time and the availability of comparisons with other previous scaffold-based research in which a simple scaffold design was used [40]. Additionally, due to their relatively small size, rapid manufacturing of such structures was possible, providing 120 3D scaffolds of all cubic configurations for the experiments. Fifty μm pores with twenty-five μm threads were used as the standard for the basic scaffold. Other basic scaffolds with quadratic pores of 40 μm, 55 μm, 80 μm, and 100 μm were employed later to test how pore size variation might influence the printing properties and cell response. The threads for these scaffold pores were scaled accordingly to maintain the ratio between the pores and threads at as close to ∼2:1 as possible. However, a cubic-pore scaffold design is only suitable for finding the right pore size for cell growth rather than mimicking the ECM. Therefore, to accommodate cell growth in a more tissue-like environment, the second design was created. This was a 1 mm diameter spheroid-like structure with a gyroid-shaped internal architecture with embedded microchannels (Figure 3b). The effective pore size in this model was 130 µm. This type of architecture is more applicable to the secundum naturam paradigm than the strict architecture of a cubic scaffold. In this manuscript, it is referred to as a spherical scaffold. Overall, 12 spherical scaffolds were made.

**Figure 4:**
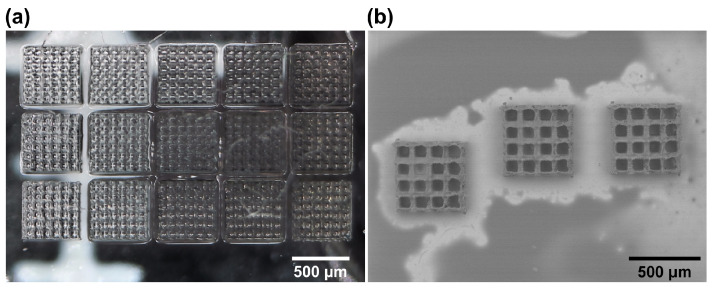
(**a**) Optical image of an array of 15 basic scaffolds with 50 μm pores, which were used for the majority of the experiments. (**b**) SEM image of basic scaffolds with 80 μm pores, showing good structural discrepancies and slight surface roughness for better cell adhesion.

With the 3D models ready, 3D printing of the basic scaffolds commenced. The microstructures could be handled manually for cell experiments. The scaffolds were printed on a quadratic glass plate (26 × 26 × 1 mm). None of the fabrication times for the cubic scaffold designs used were influenced by the choice of polymer and photoinitiator mixture (OrmoClear or PEGDA with 1% *w*/*v* Igracure 2959), nor were the slicing and hatching parameters, and they were in the order of 20 min per scaffold at a 1 cm/s translation velocity. It was shown that they can be produced in large quantities with adequate repeatability (Figure 4a). As a quantitative measure of reproducibility and quality, we chose the size deviations in the structure from the 3D model, as cross-determined using the optical microscope and SEM. The average shape deviation of these structures averaged at 5 μm, or around 1% of the overall size. This is to be expected due to the shrinkage of the material during the printing and developing process [41,42,43]. Nevertheless, the deviations were minuscule and had a minimal impact on the mechanical stability of the structures or shape fidelity, as evident from the SEM analysis (Figure 4b). Additionally, the printing parameters used (slicing and hatching steps of 5 μm) allowed the structures to have an outer surface roughness in the range of around 1 μm RMS. This was a deliberate decision, as TPP-made scaffolds with rough surfaces have been shown to facilitate cell adhesion and growth [44]. They were also beneficial with regard to faster printing times. Overall, the structures showed no tendency to break or shrink additionally over time, which are two main qualitative criteria based on which such structures are judged and can be easily determined. Thus, mechanical quality and shape fidelity were considered sufficient for the biological experiments.

**Figure 5:**
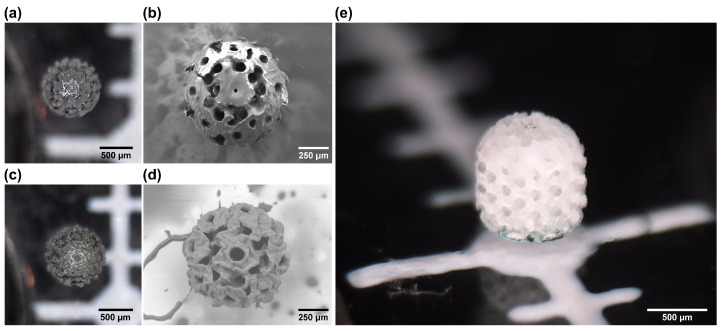
Top-down optical and SEM images of spheroid scaffolds made out of OrmoClear (**a**,**b**) and PEGDA (**c**,**d**). (**e**) An optical image of an OrmoClear spheroid scaffold from an angle. Clear and well-defined 3D pores are visible.

Spheroids were fabricated next. Here, it is important to note that the scaffold height of 1 mm exceeded the free working distance of ∼0.55 mm of the applied 20× 0.8 NA objective lens. Despite this, owing to the usage of the material vat with a submerged substrate which was lifted away from the focusing optic after each layer, printing objects with a height higher than the free working distance was made possible. Additionally, as one might expect, the printing time here was around 4 times longer and reached around 1.5 h. This can be tied to the bigger size of the structure, as well as the relatively higher infill rate. The same printing parameters were used as in the case of the basic scaffolds. However, in this case, there were some additional differences between the OrmoClear and PEGDA scaffolds. While optical microscope characterization yielded very similar images (Figure 5a,c), the SEM analysis showed that the surfaces between the two materials were somewhat different (Figure 5b,d). The OrmoClear structure ended up being a lot smoother, with its surface roughness being below 500 nm RMS. This can be explained by the interplay between the offset of the layers in different slices, allowing the formation of a more uniform surface, even with a relatively high slicing step. This phenomenon is well known in microoptics fabrication [45]. Despite this, the 3D architecture in such scaffolds was very well defined (Figure 5e). In the case of PEDGA, the structure ended up having a surface roughness a lot closer to that of the basic scaffolds. Also, there were some minor deviations from the 3D models overall in a range of up to 10 μm. This can be explained by the substantially lower viscosity of PEGDA (0.101 ± 0.03 Pa·s [46] for PEGDA vs. 1.5 ± 0.3 Pa·s for OrmoClear [38]), allowing a lot more motion of the material in the vat during printing. In the context of this work, it was considered that said deviations were acceptable, and such PEGDA spheroids were used in the in vitro experiments. However, if a smoother surface is needed, the mechanical motion of the sample in the material vat should be minimized or eliminated by applying hybrid linear stage-scanner [47] or pure scanner [48] positioning. Finally, in comparison to the cubic scaffolds, the spherical ones showed a much higher degree of mechanical resilience because none of the spherical scaffolds broke down during their removal from the glass substrate, while approximately 30% of the cubic scaffolds broke during the removal procedure. In other words, a higher fill ratio, an inherently stronger gyroid internal structure, and overall thicker threads are desirable assets, as long as their bulkiness does not exceed the size ranges needed for cell cultivation.

### 3.2. Three-Dimensional Cell Culture Optimization and Characterization

**Figure 6:**
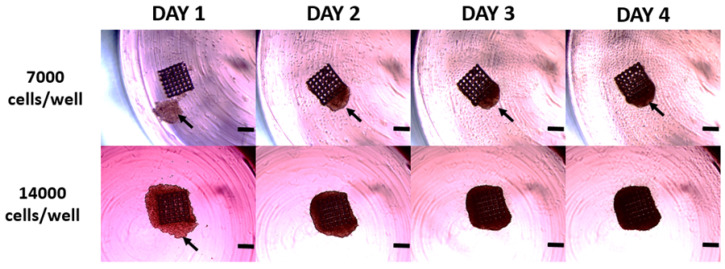
Representative images of the ovarian cancer cell line SKOV3 3D cell culture grown in 96-well plates at different seeding densities between days 1 and 4. White arrow: ovarian cancer cells. The scale bar represents 300 μm.

Two human ovarian cancer cell lines were tested for their ability to form reproducible 3D cell cultures using 3D-printed scaffolds. Said cell lines exhibit a different genetic background, morphology [49], and ability to form 3D cell cultures in a scaffold-free environment. To enable 3D cell structure formation using the printed scaffolds, we used 96-well round-bottom plates covered with agarose to ensure low attachment and prevent cell adhesion to the well bottom. This setup promoted cell–cell and cell–scaffold contact, allowing for 3D cell structure formation within a day. Initially, we determined the optimal seeding density, ranging from 7000 to 14,000 cells per well (Figure 6). The 3D cell cultures were observed every day and photographed. As shown in Figure 6, 7000 cells per well were insufficient to evenly cover the basic 500 × 500 × 250 μm size cubic scaffold and ensure uniform cell growth. In contrast, 14,000 cells per well resulted in even scaffold coverage, ensuring structured 3D cell culture formation. Consequently, we optimized the seeding density to 14,000 cells per well, which was used next with the different 3D-printed scaffolds described in Section 3.1.

### 3.3. Cell Growth and Proliferation Using Different 3D-Printed Scaffolds

**Figure 7:**
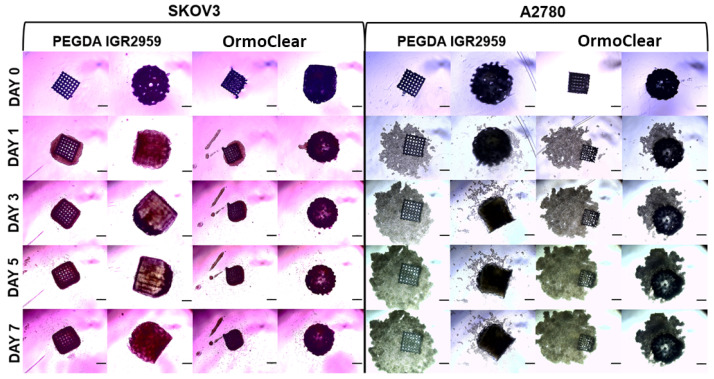
Representative images of the ovarian cancer cell line SKOV3 and A2780 3D cell cultures grown on different scaffolds between days 0 and 7. The scale bar represents 300 μm.

The ovarian cancer cell lines A2780 and SKOV3 were seeded at 14,000 cells per well on both the cubic/basic and spheroid scaffolds made from PEGDA with the IGR2959 photoinitiator or OrmoClear. The 3D cell cultures were maintained and observed for 7 days. Cell behavior, growth, and proliferation were evaluated daily, and the 3D structures were photographed (Figure 7). Here, we have to note that degradation of the scaffolds was not evaluated, as for both OrmoClear and PEGDA, it should not occur for such a short 7-day study [50]. On the first day, all the cells present in the culture had migrated toward the scaffold, and cell adherence was observed. As shown in Figure 7, the cells from both cell lines migrated toward the scaffolds regardless of the printing material used. The SKOV3 cells attached to the scaffold regardless of its shape or material, forming compact 3D cell structures. By day 7, cell proliferation was evident, with cells completely covering the scaffold. Similarly, the A2780 cell line migrated towards the printed scaffolds, though these cells formed looser 3D structures. They grew both in attachment to the scaffold and independently in proximity to it. However, this cell line formed better 3D cell structures with the spheroid scaffolds. The cubic scaffolds were not the optimal choice for the A2780 cell line when trying to achieve dense 3D cell structures. In conclusion, cell growth and proliferation were not affected by the scaffold material or shape. The cells were consistently attracted to and migrated towards the scaffolds, where they attached and grew, forming 3D cell structures.

**Figure 8:**
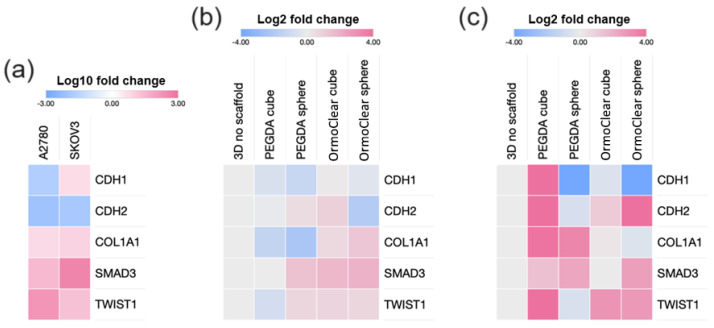
Ovarian cancer cell line SKOV3 and A2780 3D cell culture gene expression represented as heat maps: (**a**) gene expression comparison between the cell lines cultured in 3D cell culture conditions. (**b**) Gene expression comparison of SKOV3 cell line when cells were cultured without and with different scaffolds made from PEGDA or OrmoClear in cubic/standard and spheroid shapes. (**c**) Gene expression comparison of the A2780 cell line when cells were cultured without and with different scaffolds made from PEGDA or OrmoClear in cubic/standard and spheroid shapes.

On day 7, the 3D cell cultures were collected for gene expression analysis. Five genes—*CDH1*, *CDH2* [51], *COL1A1* [52], *SMAD3* [53], and *TWIST1* [54]—related to cell adhesion, migration, and extracellular matrix (ECM) organization [55] were analyzed (Figure 8). The *CDH1* gene encodes E-cadherin, while the *CDH2* gene encodes N-cadherin. Both proteins are essential for cell–cell adhesion and the epithelial–mesenchymal transition (EMT) [56]. *TWIST1* encodes a transcription factor that regulates the process, promoting the downregulation of *CDH1* and rgw upregulation of *CDH2*, where epithelial cells lose their cell–cell adhesion properties (mediated by E-cadherin) and gain migratory and invasive properties (mediated by N-cadherin) [57]. *COL1A1* (Collagen Type I Alpha 1 Chain) is a major component of the extracellular matrix, providing structural support to tissues and playing a role in cell signaling. *SMAD3* is involved in TGF-β signaling, which regulates extracellular matrix production and the EMT [58]. Therefore, the role of *CDH1*, *CDH2*, *COL1A1*, *SMAD3*, and *TWIST1* in cell–cell adhesion, ECM production, and signaling pathways may be integral to the success of tissue engineering strategies.

First, we compared the gene expression in the scaffold-free 3D structures of A2780 and SKOV3 (Figure 8a). The high expression of *CDH1* in the SKOV3 cell cultures compared to A2780 aligns with SKOV3 cells’ ability to form uniform 3D cultures even in the absence of scaffolds. Next, we analyzed the gene expression in the scaffold-free SKOV3 3D cell cultures and compared it to that in the cultures grown with scaffolds made using different materials and shapes (Figure 8b). The results revealed minimal changes in gene expression (within a two-fold threshold), indicating that the gene expression was similar both with and without scaffolds. However, there was a slight increase in the expression of *COL1A1*, *SMAD3*, and *TWIST1* when the SKOV3 cells were grown on the OrmoClear printed scaffolds. Overall, there were no substantial differences in the SKOV3 3D cell structures regardless of the scaffold material or shape.

For the A2780 cell line, which usually does not form tight 3D cell structures, we observed a substantial increase in gene expression in the 3D cultures grown on scaffolds (Figure 8c). Scaffolds made from PEGDA with the IGR2959 photoinitiator increased *COL1A1* and *SMAD3*’s expression, while the basic scaffolds also increased *CDH1* and *CDH2*’s expression. The scaffolds made from OrmoClear increased *TWIST1* and *CDH2*’s expression while downregulating *CDH1*. Interestingly, all the spheroid-shaped scaffold 3D cell cultures showed decreased expression of *CDH1*, regardless of the printing material used. The presence of scaffolds enhanced the gene expression associated with 3D cell culture formation in the A2780 cell line. In summary, culturing the A2780 and SKOV3 cell lines on scaffolds revealed significant differences in the gene expression responses. The SKOV3 cells, which naturally form uniform 3D structures even without scaffolds, showed minimal changes in gene expression when scaffolds were introduced. In contrast, the A2780 cells, which typically do not form well-defined 3D structures independently, exhibited substantial changes in gene expression when cultured on scaffolds. Specifically, scaffolds significantly enhanced the 3D-growth-related gene expression profile in the A2780 cells, providing a valuable means to improve their 3D structure formation ability.

### 3.4. Three-Dimensional Cell Cultures Grown on Scaffolds with Different Pore Sizes

**Figure 9:**
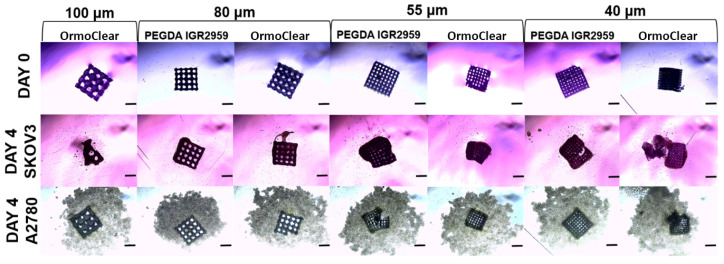
Representative images of the ovarian cancer cell line SKOV3 and A2780 3D cell cultures grown on basic scaffolds with different pore sizes ranging from 100 to 40 µm. Images represent 3D cell culture at day 4. The scale bar represents 300 μm.

The cancer cell lines A2780 and SKOV3, as previously described, were seeded on cubic/basic scaffolds with different pore sizes ranging from 100 to 40 µm. The cells were grown in culture for 7 days. The 3D cultures were observed and imaged every second day. Cell migration towards the scaffold was visible on the first day, as all the cells present in the culture migrated towards and attached to the scaffold. As previously mentioned, no visible differences were observed regarding the scaffold material (Figure 9). The scaffolds with a pore width of 100 µm made from PEGDA with the IGR2959 photoinitiator broke down during the detachment process, making them unsuitable for the 3D cell cultures. In contrast, the scaffolds of the same pore size made from OrmoClear remained intact and were successfully used. The SKOV3 cells attached well to all the scaffolds, forming 3D cell structures regardless of their pore width, as shown in Figure 8. By day 4, the best 3D cultures were observed with a scaffold pore size of 55 µm, as cells fully covered the scaffold, indicating that this pore size was ideal for the SKOV3 cell line. For the A2780 cell line, although all the cells migrated to the scaffold, better attachment was observed with a pore size of 40 µm. However, not all the cells were attached, and some remained in close proximity to the scaffold. These results suggest that for the A2780 cell line, even scaffolds with a smaller pore size could be used to achieve optimal results and create uniform 3D cell structures.

## 4. Discussion

Tumor models have allowed scientists to make significant progress in cancer research. However, several capability gaps are remaining, limiting the effectiveness and accuracy of the current in vitro models. With this study, the shortcomings in 3D culture formations were tackled. Close to 150 scaffolds of various designs were produced to accommodate all the experiments presented in this work. Such a high number of structures with varying internal geometries could be produced in a reasonable amount of time owning to TPP being a relatively hands-free approach with easily exploitable mass customization potential [59]. However, the presented throughput, while adequate, still needs to be significantly improved to accommodate the expected growth of the field. The application of extremely fast scanning could be an option [60]. On the other hand, due to the pulsed nature of the femtosecond lasers used in TPP, there is a hard fundamental limit on the translation velocity at around 100 m/s [44]. Thus, a more advanced solution is needed to surpass this. Beam shaping has been shown as a natural progression for a further increase in TPP’s throughput. So far, the demonstrations include multiplex printing via focal point arrays [61], layer-by-layer [62] printing, and voxel size/shape manipulations [63]. Understandably, all these approaches have their advantages and disadvantages, with very limited usability in commercial TPP systems. However, beam shaping seems to be the next natural step that would allow us to go beyond what is currently possible, even with the fastest galvo-scanner systems. Here, spheroid scaffolds even offer some relative simplifications to the process, such as the resolution requirement not being as strict at ∼1 μm. Also, spheroid scaffolds do not need to be bigger than ∼mms in overall size. Therefore, with these technical simplifications in mind, there should be future works aimed at this particular avenue of research with the primary aim of producing spheroid scaffolds in seconds.

In this study, different in vitro human ovarian cancer lines (SKOV3 and A2780) were used for the scaffold seeding. The microstructures fabricated showed great biocompatability with both cell lines that were used. We found that both cubic and spheroid scaffolds are suitable for cell growth, as they promote favorable cell adhesion and morphology, as well as support. It was determined that the suitable quantity for scaffold seeding was 14,000 cells per well, which ensured great coverage of the scaffolds. Other studies have shown very minimal scaffold coverage with cells. However, scaffold-based models should ensure that 3D cultures are formed with the essential quantity of cells [35,40,64]. We designed scaffolds with two different types of architectures to see which one mimicked the ECM better. Both ovarian cancer cell lines adhered to all the scaffolds no matter what kind of material they were made of. Easily forming 3D cultures, SKOV3 cells do not show any differences between different types of scaffolds. However, considering 3D culture formation, the spheroid-type scaffolds were preferable when comparing the A2780 cell growth between different types of scaffold designs and material bases. The A2780 cell line does not form 3D cultures as easily as the SKOV3 line. Thus, the differences between the designs were best seen with this cell line. The cubic scaffolds were not suitable for this kind of cell since their geometrical properties were not complex enough. However, a cubic scaffold may become handy when determining what kind of complexity cells require—for the A2780 cell line, a cubic scaffold with a smaller pore size was preferred over a cubic scaffold with bigger pores. This can be explained by the complexity of the architecture and more support for cells to adhere since there is a greater surface to attach to [65,66]. Further studies to elucidate the scaffold’s complexity and cell growth are required.

To achieve an even broader understanding of how scaffolds impact cell growth, the gene expression evaluation was performed. As a control, we used scaffold-free 3D structures of both ovarian cancer cell lines. The SKOV3 cells’ gene expression profile, which consisted of the genes *CDH1*, *CDH2*, *COL1A1*, *SMAD3*, and *TWIST1*, showed that there were minimal changes in expression. The slight increase in *COL1A1*, *SMAD3*, and *TWIST1* in the SKOV3 cells grown on the OrmoClear printed scaffolds can be explained given that OrmoClear may hinder cell–cell adhesion and provoke the expression of proteins that are required for cell support. Nevertheless, to test this, more studies are required, and no previous research has shown this. The SKOV3 line showed that there were no major differences between the scaffold architecture types, and complexity is not required for this cell line. On the other hand, the A2780 cell line showed substantial changes in gene expression when it was grown on spheroid scaffolds. The materials from which the scaffolds were fabricated also showed big differences—compared to A2780 cultured on spheroid scaffolds of PEGDA 700 with 1% *w*/*v* IGR2959, for A2780 cultured on OrmoClear spheroid scaffolds, the gene expression was elevated. One gene, *CDH1*, was downregulated. This gives us a valuable understanding and an impactful model for culturing cell lines that fail to form 3D cultures on their own. The downregulation of *CDH1* can be explained by the scaffold usage since the cells do not require intense cell–cell adhesion when a scaffold is introduced as a support for adhesion. *CDH1* encodes E-cadherin—the membrane protein responsible for cell-to-cell adhesion [67]. The upregulation of this gene in the SKOV3 scaffold-free 3D culture shows how this cell line can easily form 3D cultures. A scaffold-based system may be a great solution for the kinds of cells that have low expression of cell–cell adhesion-regulating genes. The sophisticated architecture plays a key role in enhancing the area for cell support. However, the scaffold models presented here have some limitations, as they have not yet reached a level of complexity that allows us to fully mimic the ECM. Further optimization of our models therefore implies the reconstruction of a specialized microenvironment by incorporating more complex structures such as vascular networks, immune system elements, and different types of cells (e.g., endothelial cells, fibroblasts, etc.). In addition, more sophisticated hydrogels with bioactive molecules will improve the personalized usage of scaffolds for various research purposes.

Considering the versatility of TPP when it is applied to 3D spheroid scaffold fabrication, more intricate designs for complex tissue engineering can be fabricated. For instance, we would like to draw additional attention to the horizontal threads present in the models printed during this work. Conventional 3D-printing wisdom would dictate that such overhangs would require additional support when printing in liquid resin. Otherwise, there may be a potential risk of the structure collapsing during the printing process. Nevertheless, due to the very small sub mm size of these threads, even support-free printing yielded the required structures. This is one of the many advantages of TPP, i.e., that complex structures with clear pronounced overhangs can be printed directly without supports if the material used has a high enough viscosity [17]. Therefore, it is clear that TPP provides us with a unique opportunity in terms of bringing standardization to 3D cell culture generation. With optimization of the fabrication process, 3D culture scaffolds can bring reproducibility to spheroid generation and ensure their adaptability for drug testing [39]. Together with a more sophisticated scaffold architecture, materials in the forms of bioinks need to be developed to support cell adhesion, growth, and differentiation [68,69]. It is important to note that TPP offers unmatched flexibility in terms of the biomaterial selection [27]. Even more importantly, this technology can combine different material properties or even materials within the same 3D structure. It can be achieved either using an intensity-modulated crosslinking degree [70] or direct multi-material printing [71]. Multi-material structures very similar to those tested in this work and combining hybrid organic–inorganic materials and PEGDA have already been demonstrated in the previous literature [72]. The scaffold architecture also plays a key role in determining the structure’s integrity and mechanical strength [73,74]. TPP’s capabilities can also be leveraged here to a great extent, as it offers a true free-form fabrication capability in the micrometer range. The scaffolds that showed low structural integrity (models with pore sizes of 100 µm) were unsuccessful in the biological evaluation and would be susceptible to damage with long-term usage. To optimize the pore size and the structure of the scaffolds themselves, we need to consider the physical parameters of the cells to create the best possible environment for cell growth. This study showed that more complex scaffold architectures are preferable for mimicking the ECM, especially with cell culture lines that do not form 3D structures in vitro. On the other hand, the architectural simplicity of scaffolds can be handy for cell count determination or establishment if the cell culture is able to form a 3D culture independently. TPP provides us with a unique opportunity in terms of bringing standardization to 3D cell culture generation. Due to the specialties of TPP, 3D spheroid scaffolds can be fabricated in big batches, which would allow for wider industrialization. With optimization of the fabrication process, 3D culture scaffolds can bring reproducibility and higher accuracy within the conducted studies to spheroid generation and ensure their adaptation for drug testing. The wide usage of scaffold-based spheroid systems could elevate and optimize drug testing by lessening the usage of animal models and shorten the period of time required to characterize potential drugs in the preclinical stages of drug development trials. Considering this, further research should be carried out, with a focus on testing various scaffold designs and applying them to more complex tissue engineering.

## 5. Conclusions

In summary, TPP was applied for the microfabrication of scaffolds made of biocompatible photopolymers. This work demonstrated that a high count of microstructures destined for 3D cell formation can be produced in a short time. The insights gained from this study highlight the potential of TPP-fabricated scaffolds in cancer research. The geometric properties of the scaffolds can be easily adjusted for the specific needs of 3D cell culture applications. OrmoClear and PEGDA with IGR2959 were used for the fabrication of the scaffolds. Our biological evaluation showed there was no significant difference in cell growth between the OrmoClear and PEGDA scaffolds. However, PEGDA was preferable in the case of spheroidic scaffolds for the A2780 cell line. The scaffold architecture type showed differences for the A2780 cell line—spheroid scaffolds were a hit for forming 3D cultures. The A2780 gene expression evaluation showed that the gene expression for *CDH1*, *CDH2*, *COL1A1*, *SMAD3*, and *TWIST1* was altered. Meanwhile, the SKOV3 cell line’s growth was disturbed neither by the scaffold architecture nor by the material choice. Still, the gene expression evaluation showed that the SKOV3 cell line’s genes were altered too. This has a meaningful impact in terms of showing how 3D cell culture is different from 2D culture, and even small gene expression alterations can have an impact on experiments’ precision. Future research could explore the usage of other photopolymerizable materials with non-cellular components of the ECM (e.g., growth factors) and refine the scaffold designs further to enhance cell growth and mimicry of the tumor microenvironment. Ultimately, this research could contribute to the development of more effective cancer research models and improve the accuracy of preclinical drug testing models.

## Figures and Tables

**Table 1:** The primers used for gene expression evaluation with real-time qPCR.

Gene	Forward Primer (5′–3′)	Reverse Primer (3′–5′)
*RPL13a*	GCCATCGTGGCTAAACAGGTA	GTTGGTGTTCATCCGCTTGC
*CDH1*	CGAGAGCTACACGTTCACGG	GGGTGTCGAGGGAAAAATAGG
*CDH2*	TCAGGCGTCTGTAGAGGCTT	ATGCACATCCTTCGATAAGACTG
*COL1A1*	GAGGGCCAAGACGAAGACATC	CAGATCACGTCATCGCACAAC
*TWIST1*	GTCCGCAGTCTTACGAGGAG	GCTTGAGGGTCTGAATCTTGCT

## Data Availability

The data are available upon request from the authors.
